# Unmasking Bullying: A Cross-Sectional Study on Its Prevalence and Impact Among School-Aged Children

**DOI:** 10.7759/cureus.76788

**Published:** 2025-01-02

**Authors:** Sheetal Kotgirwar, Jitesh Patil, Sunita Athavale, Rekha Lalwani

**Affiliations:** 1 Anatomy, All India Institute of Medical Sciences, Bhopal, IND

**Keywords:** abuse, anxiety, bullying, depression, government school, school bullying, victimization

## Abstract

Background: Bullying among school-going children is a significant public health issue that impacts mental and physical well-being. This cross-sectional study aims to assess the prevalence and factors contributing to bullying in school-going children. The study seeks to give data for policymakers and parents, as it can inform initiatives aimed at improving the mental health of bullied children.

Material and methods: This cross-sectional study comprised 1,000 students as study participants between the age group of 11 to 19 years. Both private and government schools were selected. Three different prevalidated questionnaires were utilised to gather data on the number of bullied students, their psychological health, and their social interactions. The data collected were quantitatively analysed to ascertain the number of students experiencing bullying and the psychiatric issues that may have arisen as a result.

Results: The prevalence of bullied students was 399 out of 856 (46.73%), which was similar in government and private settings. The proportion of bullied students reduced with higher age and grades. The mean age of bullied students was 14.27±1.68 years. The majority of students reported good self-esteem. However, about a quarter of the students reported clinically significant anxiety.

Conclusion: The findings of this study clearly indicate that bullying is alarmingly prevalent among school-aged children. The data suggest a strong correlation between bullying involvement and behavioural problems among adolescents. This underscores the urgent need to investigate the causes of bullying further and to implement targeted interventions within schools to reduce its prevalence.

## Introduction

The United Nations Children's Fund (UNICEF) published a global status report on school violence and bullying. This report highlighted the scope of bullying in school-going children and reported the prevalence of bullying ranging from 10% to 65% in different countries. It calls bullying a pattern and not just an incident that impacts the victim, bully, and other students physically, emotionally, and educationally [1].

In India, the ramifications of bullying profoundly impact the physical and mental well-being of children, often with tragic outcomes. A concerning trend reveals that bullying predominantly affects children and adolescents from disadvantaged backgrounds, exacerbating their struggles as they navigate the school environment. Statistics indicate that six out of 10 children and teenagers in India experience bullying, with a pronounced effect on those from poorer communities. For instance, Madhya Pradesh alone accounts for nearly 6% of bullying cases in the country [2].

Numerous studies have established a link between childhood or adolescent bullying and the subsequent development of mental health issues, including depression, anxiety, and suicidal ideation [3]. Victimisation has emerged as a critical factor undermining children's psychological health, frequently originating in the school setting but often going unrecognised [4-10]. Given that adolescents spend a considerable amount of time in school, research has increasingly focused on the educational context as a crucial environment for mental health outcomes [11,12]. 

The consequences of bullying extend to various psychiatric disorders, such as depression, post-traumatic stress disorder, anxiety disorders, substance abuse, and suicidal behaviour [13]. While the negative health and psychosocial impacts of bullying are well-documented, research specifically addressing these issues in India remains limited [4, 13-16]. To address this gap, the present study aims to determine the prevalence of bullying, explore the dynamics of bullying in school-going children, and seek to understand the underlying factors.

## Materials and methods

Study design and setting

A cross-sectional study was conducted to obtain data on bullying. The study was conducted in government and private schools across Bhopal after obtaining approval from the Institutional Human Ethical Committee of All India Institute of Medical Sciences, Bhopal, India, for the study (LOP no: AIIMS/BPL/IECSR/JAN/23/STS/07 dated 24 Aug 2023).

Study population

The cross-sectional study comprised students ages 11 to 19 who were enrolled in classes sixth through twelfth. Since adolescents deal with many issues throughout this stage of life, they are the study's target population.

Sample Size

A multiple-stage random sampling technique was used to select Bhopal's government and private schools. Originally, 1,000 students were registered to participate in this study; a sample size of 856 students was used, and 144 students were excluded from the study as they were not willing to participate.

Sampling technique

Both private and government schools were selected; each section comprised 40 students. Therefore, two sections per class in each school had to be chosen to reach the sample size. They were selected at random from school. In every government and private school that was selected, two sections of a single grade were chosen at random, ensuring that every class was represented in the sample. Every student's data from the chosen portions is included in the research.

Data collection methods and questionnaire

Data were collected anonymously through a physical questionnaire form filled out by them in their regular classes.

Consent Form

First, permission and consent were taken from students, and they were explained about the confidentiality of this research study.

Demographic Data

It included information about students, including their name, grade, gender, age, type of school, siblings, parents’ occupation, and education.

Peer Relations Questionnaire (PRQ)

It was used as a self-report measure for bullying. There were three subscales (victim, bully, and pro-social) and 12 items in total. A four-point Likert scale was used to rate the responses. The greater frequency of each behaviour tested was indicated by higher scores. A child was considered to be bullied if their victim scale score was more than 10 (Appendix A) [17].

Rosenberg Self-Esteem Scale (RSS)

This scale was used to assess self-esteem. Ten elements make up the scale: five are positive remarks, and five are negative statements. Typically, the RSS is graded using a Likert scale. Students have the option to select from four possible answers: strongly disagree, disagree, agree, and strongly agree. A minimum score of 10 and a maximum score of 40 were assigned to the RSS. In other instances, the range may vary from 0 to 30 based on the study and the way the response categories were added and classified. Students scoring more than 15 are classified as having strong self-esteem, while those scoring less than or equal to 15 are classified as having poor self-esteem (Appendix B) [18].

Screen for Child Anxiety-Related Disorders (SCARED) Scale

It was used for measuring anxiety in children. It involved 41 items that assess a child’s recent anxiety symptoms. Participants respond on a three-point Likert scale of 0 (not true or hardly ever true), one (somewhat or sometimes true), or two (very true or often true). A score of 25 and greater than 25 was suggested to be indicative of the presence of clinically significant anxiety (Appendix C) [19].

## Results

Table 1 shows the descriptive statistics of the demographic details of the study population. The participation of government schools was slightly higher than that of private schools.

**Table 1:** Category-wise distribution of the demographic details of the study population

Demographic details		Frequency (percentage)
Type of school	Government	476 (55.60)
	Private	380 (44.40)
Gender	Male	477 (55.72)
	Female	379 (44.28)
Mean age of the students in years (Mean ± Std. Deviation)	14.33 ± 1.79	

Table 2 shows the distribution of the study population based on grades in school. The participants belonged to almost all grades, from middle to higher secondary classes.

**Table 2:** Distribution of the study population based on different grades in school

Grade	Frequency	Percentage
6	86	10.04
7	143	16.73
8	132	15.42
9	204	23.83
10	121	14.13
11	129	15.07
12	41	4.78

**Figure 1:**
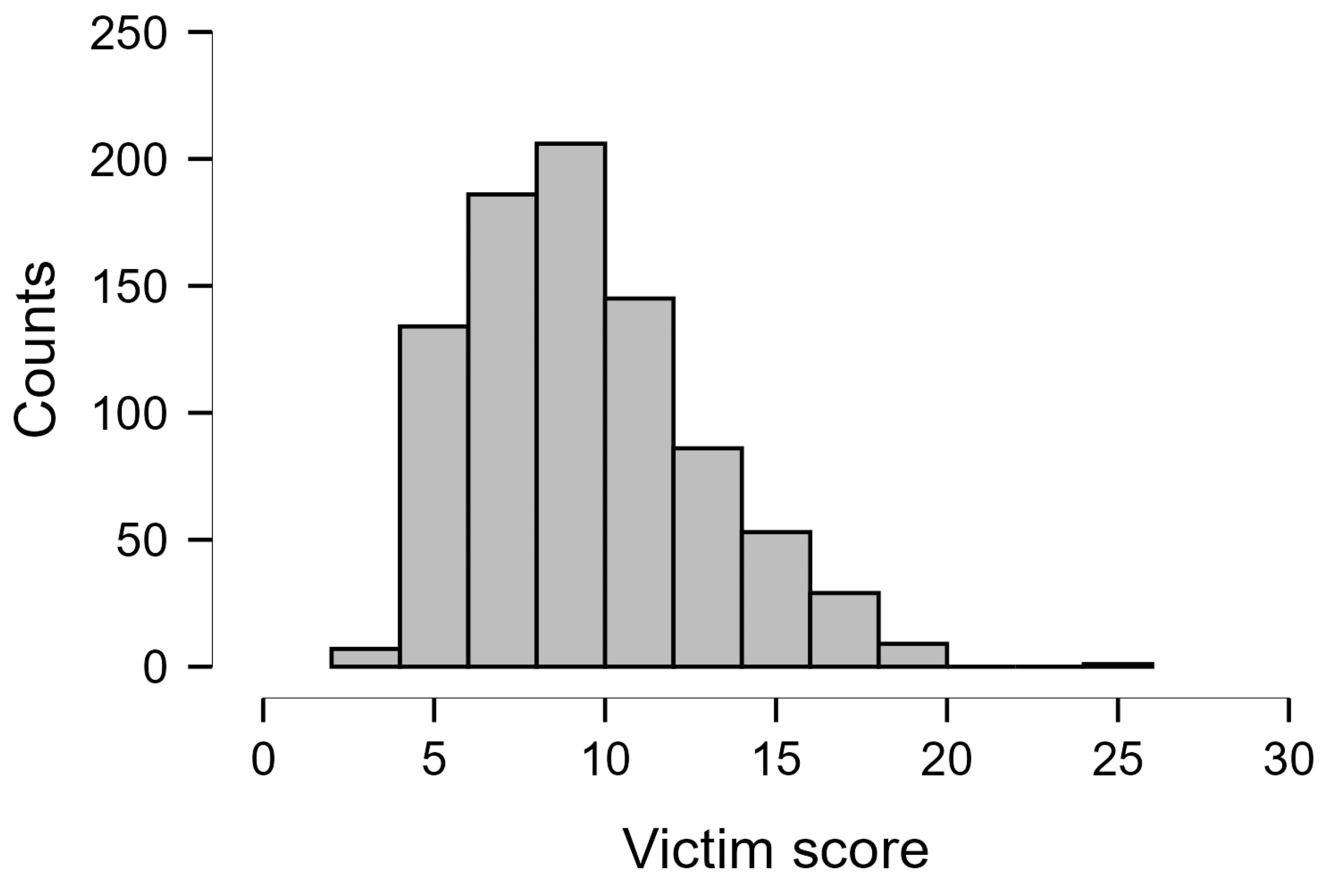
Distribution of the victim scores in the study population

Figure 1 shows the distribution of victim scores. The scores ranged from four to 20 on a scale of four to 20. These scores indicated perceived victimhood of being bullied. A score of 10 or more was labelled as being bullied. According to this scale, 399 (46.73%) out of 856 students were bullied.

**Figure 2:**
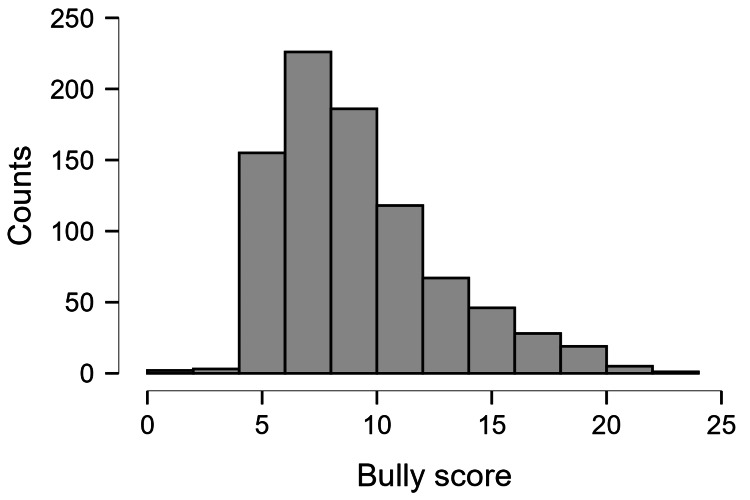
Distribution of the bully scores in the study population

Figure 2 shows the distribution of the bully scores. This score indicates the traits of students who bully others. The scores ranged from six to 24. A higher score indicated the tendency of the student to bully other students. A score of 12 or above was considered as students bullying others. The number of students who bullied others was proportionately less than those who were bullied. 

**Figure 3:**
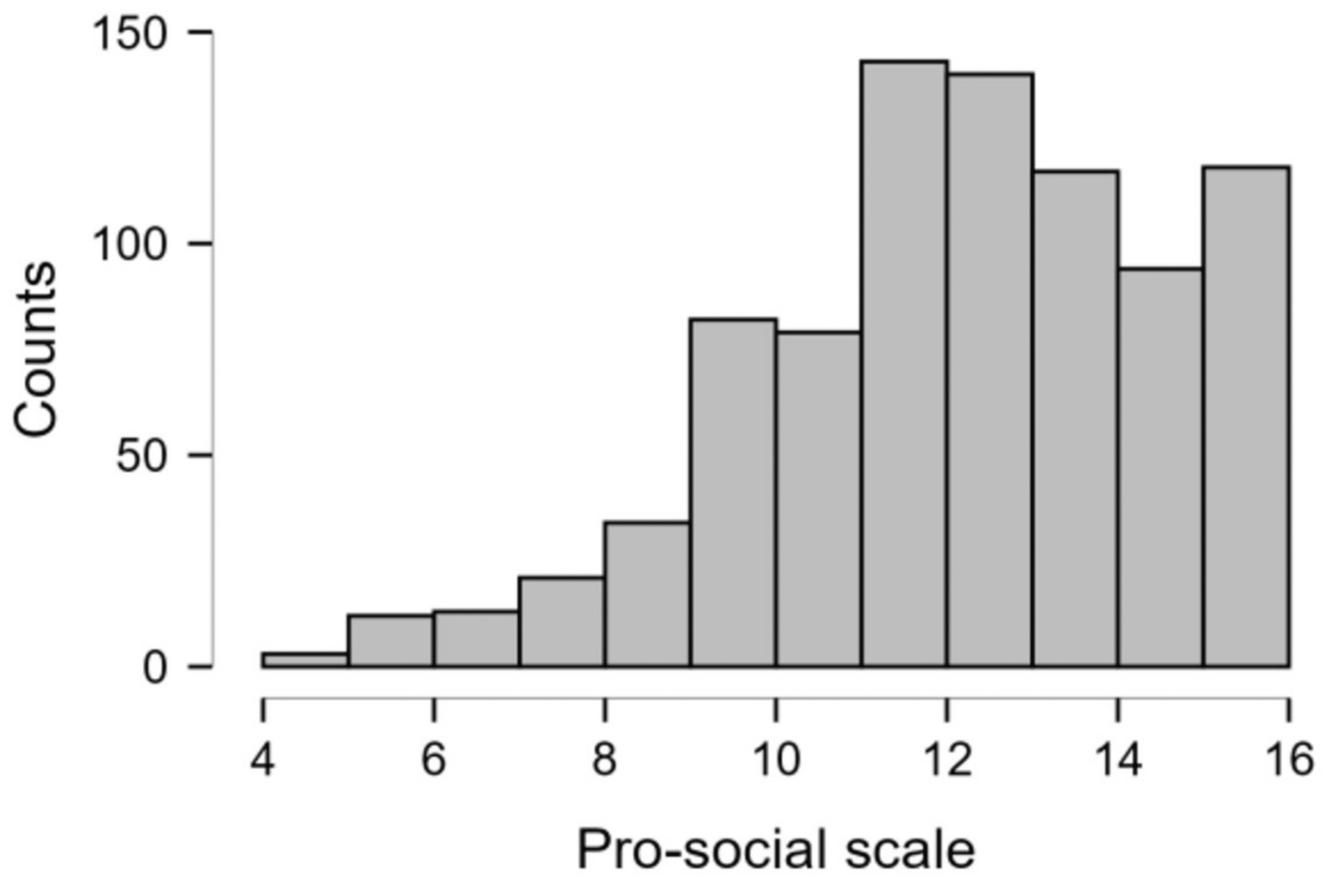
Distribution of pro-social scores in the study population

Figure 3 shows the distribution of the pro-social scores of the students. This scale indicates the social behaviours of the student on a scale of four to 16. Most students had scores of more than eight on this scale, indicating that they exhibited normal social behaviour. 

**Figure 4:**
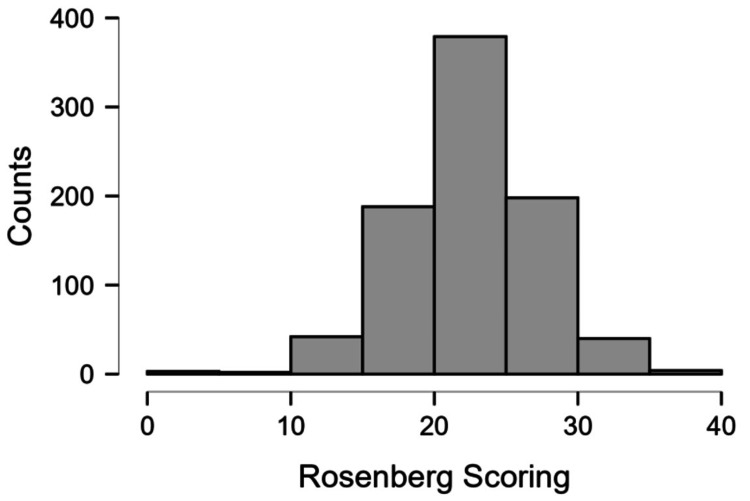
Distribution of Rosenberg score in the study population

Figure 4 depicts the scores on the Rosenberg scale. Most of the students exhibited strong self-esteem (scores 15 or above).

**Figure 5:**
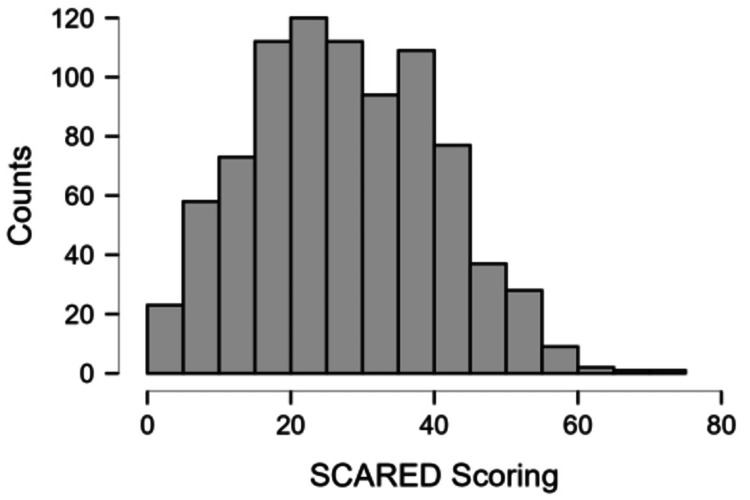
Distribution of the SCARED score in the study population SCARED: Screen for Child Anxiety-Related Disorders

Figure 5 shows the distribution of SCARED scores of the students. This scale indicates the anxiety levels of the students on a scale of 0-82. A score of 25 and above indicated clinically significant anxiety. 

The victim scores and the SCARED scores were significantly positively correlated (Table 3). The correlation between the grades of the students and the victim score was negatively correlated. This indicated that the students were less prone to being bullied in higher grades. Similarly, the age of the students was slightly negatively correlated with age.

**Table 3:** The correlation of victim scores with SCARED scores, age, and grades *p < .05, ** p < .01, *** p < .001 SCARED: Screen for Child Anxiety-Related Disorders

Variable	Pearson's r	P-value
Victim score and SCARED score	0.34	1.50×10^-24 ***^
Victim score and age	-0.08	0.02^*^
Victim score and grades	-0.06	0.08

## Discussion

This cross-sectional study estimated the prevalence of bullying in school-going children in sixth to twelfth grade in Indian settings. The present study observed that 399 (46.62%) out of 856 students had experienced bullying of any form. This was within the range of reports from other European nations (9% in Sweden to 54% in Lithuania) [20]. A recent meta-analysis of 80 international studies found that the mean prevalence rate of bullying participation was 35% [21]. Other high-income nations have also published prevalence rates (Australia: 18% for victims, 3% for bullies, and 5% for victims of bullying; USA: 8.4% for victims, 8.2% for bullies, and 3.7% for victims of bullying; and North America: 16.7% for victims, 15.1% for bullies, and 6.8% for victims of bullying) [20, 22]. The present study showed a higher prevalence of bullying as compared to what has been reported in the literature. The prevalence in low- and middle-income countries was in a wider range, 8% in Tajikistan and 70% in Zambia [22]. However, on the whole, the prevalence was higher in these settings as compared to the developed nations. The prevalence in India falls somewhere in between the two ends. This may be because of lower levels of education of the parents and the general atmosphere in schools where the traits of bullying go unchecked.

Consistent with past research, the results of this study demonstrate that bullying occurs in Indian schools, as neither instructors nor other adults in the school took any look at bullying that occurred at school [23,24]. Bullies are encouraged to victimise other students at schools with a positive bullying culture, and victims are likewise discouraged from reporting bullying to officials in the school.

The bullying trait is reduced with an increase in age and grades. This showed that students of lower grades were more prone to being bullied. Efforts should be made to address this problem, and specific provisions should be in place so that the psychological impact of this can be reduced for the affected students.

The students generally scored well on the Rosenmuller scale, indicating that the students had good self-esteem despite the prevalent bullying culture. Similarly, most of the students fared well on the SCARED scale, which indicates the presence of significant anxiety. However, the number of students who showed the presence of clinically significant anxiety was about a quarter of all study participants.

In the present study, the authors found that private and government schools had almost equal exposure to bullying. This was different from the recent study done in Brazil (2015), which reported that bullying victimisation was more likely to be reported in private schools than in government schools (odds ratio (OR) =1.17; 95% CI: 1.04-1.31) [23].

The insights from this study can be instrumental for school governing bodies, policymakers, and mental health professionals, providing a clearer understanding of the challenges faced by school-aged children. Addressing issues related to bullying and its psychosocial impacts is crucial for promoting better mental health outcomes.

It is imperative to raise awareness about bullying within society, encouraging children to share their experiences and seek support rather than suffer in silence. By fostering inclusive environments and positive social dynamics, we can help build a healthier future for the next generation.

Because the study is cross-sectional in nature, it has limitations when it comes to drawing conclusions about causality. All of the study's data were gathered via self-administered questionnaires. One drawback of utilising self-administered questionnaires is that some of the questions may be hard for students to grasp. In order to address this shortcoming, the authors individually went over each question with the participants before distributing the questionnaire so that students don’t find it hard to understand the questions.

## Conclusions

The findings of this study clearly indicate that bullying is alarmingly prevalent among school-aged children in Indian settings, and almost every second child reports having been bullied at some point in time. The students in middle schools in their mid-teens are more prone to getting bullied. About a quarter of students showed indications of clinically significant levels of anxiety as a result of bullying. Both government and private school students are affected equally.

## Appendices

Appendix A 

**Table 4:** The Peer Relations Questionnaire (PRQ) for children

The peer relations questionnaire (PRQ) for children [17] shows how often the following statements are true of you. To do this, tick one of the answers in front of each statement.					
S. no	Question/ statement	Never	Once in a while	Pretty Often	Very Often
1	I like playing sport				
2	I get good marks in class				
3	I get called names by others				
4	I give soft kids a hard time				
5	I like to make friends				
6	I play up in class				
7	I feel I can't trust others				
8	I get picked on by others				
9	I am part of a group that goes around teasing others				
10	I like to help people who are being harassed				
11	I like to make others scared of me				
12	Others leave me out of things on purpose				
13	I get into fights at school				
14	I like to show others that I'm the boss				
15	I share things with others				
16	I enjoy upsetting wimps				
17	I like to get into a fight with someone I can easily beat				
18	Others make fun of me				
19	I get hit and pushed around by others				
20	I enjoy helping others				
Interpretation: This questionnaire contains 3 sub-scales and several filler items. The scoring of the scales is as follows: Never = 1; Once in a while = 2; Pretty often = 3 Very often = 4; Items belonging to the scales are these: Bully Scale: 4, 9, 11, 14, 16, 17; Victim Scale: 3, 8, 12, 18, 19; Pro-Social Scale: 5, 10, 15, 20					

Appendix B 

**Table 5:** Rosenberg Self-Esteem Scale questionnaire

Rosenberg Self-Esteem Scale [18] ; Below is a list of statements dealing with your general feelings about yourself. Please indicate how strongly you agree or disagree with each statement (tick your option).					
s. no	Question	Strongly agree	Agree	Disagree	Strongly disagree
1	On the whole, I am satisfied with myself.				
2	At times I think I am not good at all.				
3	I feel that I have a number of good qualities				
4	I am able to do things as well as most other people.				
5	I feel I do not have much to be proud of.				
6	I certainly feel useless at times.				
7	I feel that I'm a person of worth, at least on an equal plane with others				
8	I wish I could have more respect for myself.				
9	All in all, I am inclined to feel that I am a failure.				
10	I take a positive attitude toward myself.				
Scoring: Items 2, 5, 6, 8, 9 are reverse scored. Give “Strongly Disagree” 1 point, “Disagree” 2 points, “Agree” 3 points, and “Strongly Agree” 4 points. Sum scores for all 10 items. Keep scores on a continuous scale. Higher scores indicate higher self-esteem.					

Appendix C 

**Table 6:** Screen for Child Anxiety Related Disorders (SCARED) questionnaire

Screen for Child Anxiety Related Disorders (SCARED)[19]; Child Version - Page 1 of 2 (To be filled out by the child) Name: Date: Directions: Below is a list of sentences that describe how people feel. Read each phrase and decide if it is “Not True or Hardly Ever True” or “Somewhat True or Sometimes True” or “Very True or Often True” for you. Then for each sentence, fill in one circle that corresponds to the response that seems to describe you for the last 3 months.				
		0 Not True or Hardly Ever True	1 Somewhat True or Sometimes True	2 Very True or Often True
1.	When I feel frightened, it is hard for me to breathe	o	o	o
2.	I get headaches when I am at school	o	o	o
3.	I don’t like to be with people I don’t know well	o	o	o
4.	I get scared if I sleep away from home	o	o	o
5.	I worry about other people liking me	o	o	o
6.	When I get frightened, I feel like passing out	o	o	o
7.	I am nervous	o	o	o
8.	I follow my mother or father wherever they go	o	o	o
9.	People tell me that I look nervous	o	o	o
10.	I feel nervous with people I don’t know well	o	o	o
11.	My I get stomachaches at school	o	o	o
12.	When I get frightened, I feel like I am going crazy	o	o	o
13.	I worry about sleeping alone	o	o	o
14.	I worry about being as good as other kids	o	o	o
15.	When I get frightened, I feel like things are not real	o	o	o
16.	I have nightmares about something bad happening to my parents	o	o	o
17.	I worry about going to school	o	o	o
18.	When I get frightened, my heart beats fast	o	o	o
19.	I get shaky	o	o	o
20.	I have nightmares about something bad happening to me	o	o	o
21.	I worry about things working out for me	o	o	o
22.	When I get frightened, I sweat a lot	o	o	o
23.	I am a worrier	o	o	o
24.	I get really frightened for no reason at all	o	o	o
25.	I am afraid to be alone in the house	o	o	o
26.	It is hard for me to talk with people I don’t know well	o	o	o
27.	When I get frightened, I feel like I am choking	o	o	o
28.	People tell me that I worry too much	o	o	o
29.	I don’t like to be away from my family	o	o	o
30.	I am afraid of having anxiety (or panic) attacks	o	o	o
31.	I worry that something bad might happen to my parents	o	o	o
32.	I feel shy with people I don’t know well	o	o	o
33.	I worry about what is going to happen in the future	o	o	o
34.	When I get frightened, I feel like throwing up	o	o	o
35.	I worry about how well I do things	o	o	o
36.	I am scared to go to school	o	o	o
37.	I worry about things that have already happened	o	o	o
38.	When I get frightened, I feel dizzy	o	o	o
39.	I feel nervous when I am with other children or adults and I have to do something while they watch me (for example: read aloud, speak, play a game, play a sport)	o	o	o
40.	I feel nervous when I am going to parties, dances, or any place where there will be people that I don’t know well	o	o	o
41.	I am shy	o	o	o
*For children ages 8 to 11, it is recommended that the clinician explain all questions, or have the child answer the questionnaire sitting with an adult in case they have any questions. SCORING A total score of ≥ 25 may indicate the presence of an anxiety disorder. Scores higher than 30 are more specific.				
